# Electrophysiology Echoes Brain Dynamics in Children and Adolescents With Tourette Syndrome—A Developmental Perspective

**DOI:** 10.3389/fneur.2021.587097

**Published:** 2021-02-15

**Authors:** Aribert Rothenberger, Hartmut Heinrich

**Affiliations:** ^1^Clinic for Child and Adolescent Psychiatry and Psychotherapy, University Medical Center Göttingen, Göttingen, Germany; ^2^neuroCare Group, Munich, Germany; ^3^kbo-Heckscher-Klinikum, Munich, Germany; ^4^Research Institute Brainclinics, Brainclinics Foundation, Nijmegen, Netherlands

**Keywords:** electrophysiology, brain maturation, Tourette syndrome, tics, development, treatment, children, ADHD

## Abstract

The development of the complex clinical picture of motor and vocal tics in children and adolescents with Tourette syndrome (TS) must be paralleled by changes in the underlying pathophysiology. Electrophysiological methods such as EEG and event-related potentials (ERPs) are non-invasive, safe and easy to apply and thus seem to provide an adequate means to investigate brain dynamics during this brain maturational period. Also, electrophysiology is characterized by a high time resolution and can reflect motor, sensory and cognitive aspects as well as sleep behavior. Hence, this narrative review focuses on how electrophysiology echoes brain dynamics during development of youngsters with TS and might be useful for the treatment of tics. A comprehensive picture of developmental brain dynamics could be revealed showing that electrophysiological parameters evolve concurrently with clinical characteristics of TS. Specifically, evidence for a maturational delay of motor inhibition related to cortico-spinal hyper-excitability and brain mechanisms for its cognitive compensation could be shown. Moreover, deviant sleep parameters and probably a stronger perception-action binding were reported. For neuromodulatory treatments (e.g., neurofeedback; repetitive transcranial magnetic stimulation, rTMS/transcranial direct current stimulation, tDCS) targeting neuronal deficits and/or strengthening compensatory brain mechanisms, pilot studies support the possibility of positive effects regarding tic reduction. Finally, attention-deficit/hyperactivity disorder (ADHD), as a highly frequent co-existing disorder with TS, has to be considered when using and interpreting electrophysiological measures in TS. In conclusion, application of electrophysiology seems to be promising regarding clinical and research aspects in youngsters with TS.

## Introduction

Tourette syndrome (TS) is characterized by motor and vocal tics, which can become apparent as early as 2–3 years of age. Usually, tics increase with a peak at about 10–12 years, but they decrease spontaneously afterwards, so that only a minority of patients (20–30%) will continue to suffer from their tics still in adulthood. Besides this motor aspect, children may report a sensory-motor urge (around the age of 9–10 years) before a tic, which might be relieved after ticcing. Also, they are able to suppress this urge and their tics, at least to withhold them for a short amount of time. This voluntary suppressibility implies a better tic control, increases by later adolescence and may be related mainly to the frontal lobe maturational course (1).

This complex clinical development of tics must evolve concurrently with a neurobiological development of tic generation. Moreover, the close association between TS and attention-deficit hyperactivity disorder (ADHD) during childhood and adolescence (about 50–60% of tic children develop also ADHD) has to be considered, when investigating the developmental brain dynamics of TS (2, 3). Also, ADHD is mainly responsible for the psychosocial problems in TS + ADHD and usually needs to be treated first. If medication is indicated, primarily stimulants are prescribed for ADHD, based on the hypo-dopaminergic pathophysiological mechanisms. For TS dopamine blockers are recommended based on the hyper-dopaminergic background.

Electrophysiological approaches are non-invasive, safe, can be applied continuously through child development in the same way and thus seem to be adequate in order to investigate the maturational brain dynamics of TS. As tools, electroencephalography (EEG), evoked potentials (EPs), event-related potentials (ERPs), transcranial magnetic stimulation (TMS) and transcranial direct current stimulation (tDCS) are available. Moreover, EEG/ERPs can be used for neurofeedback and repetitive TMS and tDCS may present other possible treatment approaches (4, 5). Electrophysiology has the advantage that it can register/influence brain activity in the millisecond range and thus represent an “online” time resolution. Hence, neuronal activation associated with a wide variety of brain states and processes (including the interaction between neural states and motor, sensory or cognitive performance) can be directly measured—even during sleep (6–9).

This narrative review focuses on how electrophysiology echoes the brain dynamics during the development of youngsters with TS. In the nearer future, one may aspire to support the clinical expert in the use of neurobiological background information for a better personalized diagnostic and treatment. On the one hand, one should be cautious while drawing firm conclusions from a particular electrophysiological finding available, so far, since usually small samples have been investigated with unclear generalizability and lack of independent replications. On the other hand, there is converging evidence regarding maturational brain dynamics of TS particularly in the motor system and aspects of comorbidity (with ADHD) as will be described in this article.

## Basic Electrical Brain Activity (Oscillations)

The evaluation of the standard EEG in children with neurodevelopmental disorders shows (by visual inspection) in several cases abnormalities (e.g., more slow waves and epileptiform discharges) [(9, 10); p. 67, (11)] reflecting a functional liability at least at the level of the cortex. For TS, this is supported by EEG abnormalities in nine monozygotic TS-twins (8–26 years), where the twin with the more severe course of illness had significantly more fronto-central theta activity (12). Also, a preliminary study in 10 children with TS [vs. healthy controls (HC)] analyzing resting-state EEG by sample entropy/multiscale entropy found for TS a statistically significant reduction of EEG complexity mainly in lower frequencies, suggesting disturbed brain connectivity (13). Similarly, when evaluating the spatial patterns of the resting-state EEG network in children (TS: *n* = 36; HC: *n* = 21), Duan et al. described a decreased intrinsic long-range connectivity between the frontal and the temporal/occipital/parietal lobes for TS (14). This finding parallels results from an fMRI-based connectivity analysis (15). Further, Hong et al. compared nine children with TS (vs. healthy controls) with EEG recordings during spontaneous expression and suppression of tics (16). Their main point was increased oscillations in the theta/beta band at fronto-central areas during tic suppression, suggesting a fronto-motor interaction of cortical oscillations.

Such oscillations (i.e., a band of periodic neuronal frequencies with a wave duration from milliseconds to several seconds) allow us to investigate neuronal connectivity as well as interactions of brain systems and their modulation as during transitional processes from rest to tic-appearance. Preliminary data suggest that two separate oscillatory mechanisms may exist: a primary quantitative “pre-post-activation” and (with a mini-delay) one in parallel, modulatory/qualitative “on-top,” processing the ongoing action by temporal neuronal synchronization of the oscillatory network in question (17). Leckman et al. suggested that in TS this network may be disturbed and driven by misguided thalamo-cortical brain oscillations (18). Usually, coherent network activity is likely to modulate sensorimotor gating as well as focused motor actions. When these networks are disturbed, as in TS, they may become dysrhythmic, leading to a loss of neuronal control and thus displaying tics. Re-gaining oscillatory control may be achieved by willfully training regions of the prefrontal cortex by applying behavioral treatments like neurofeedback as part of cognitive-behavioral-therapy (CBT) (19).

## Motor Aspects

Motor and vocal tics can be viewed as signs of hypermotoric behavior where fragments of normal movements are released because of non-successful automatic inhibitory control. The disinhibition concept of TS was recently broadly discussed by Kurvits et al. (20), who emphasized “the necessity of conceptual diversity in the scientific exploration of TS, from disinhibition and beyond.” Specifically, this holds true because different functional domains (e.g., motor, sensory, cognitive) as well as developmental considerations have to be taken into account.

A few studies in children and adolescents used TMS to investigate motor cortex excitability during development. Moll et al. (21) (TIC: *n* = 21, HC: *n* = 25) extended a study in adults (TS: *n* = 20, HC: *n* = 21) (22). The latter had found that motor threshold and peripheral motor excitability were normal in TS, while the cortical silent period (CSP), as a measure of cortico-spinal excitability (CSE) was shortened and the intracortical inhibition reduced. These abnormalities were seen mainly when tics were present in the EMG target muscle of the patients. These findings are consistent with the hypothesis that tics in TS may be primarily generated at the level of the basal ganglia, then passing through to an inhibitory impaired motor cortex and thus leading to an open tic-behavior. In the study in youngsters (ages 10–16) (21), intracortical inhibition/facilitation did not differ between TS and healthy controls which could be due to a high percentage of patients with comorbid ADHD and OCD in the adults study.

The CSP was also found to be shortened in youngsters with TS, but tic distribution (i.e., presence or absence of distal tics) did not play any role. In order to see whether this differential effect regarding tic distribution actually reflected a developmental effect, a cross-sectional analysis was conducted considering three age groups (8–11.5, 11.5–15, 15–19 years, with/without distal tics; in total *n* = 127 youngsters with tic disorders) with TMS (23). Only in the 15–19 years subgroup, was CSP shorter in patients with distal tics and confirming the results of the adults study. These findings suggest a decreased control of cortico-spinal motor networks, probably related to a larger area of the motor system in youth compared to adult patients. Cortical silent period increased with age indicating that the inhibitory processes in the cortico-spinal motor system mature to adult level around the age of 16 years. This is in parallel to the development of a better tic control at this age supporting a differential modeling of TS brain dynamics of children vs. adults.

More recently, Pepes et al. studied cortical motor excitability (i.e., resting motor threshold and MEP) in adolescents and young adults (*n* = 17; age range 11.9–21.6 years) and came to similar conclusions, namely, that MEP responses normalized during adolescence interpreting this as a developmental delay of maturation (24).

The question whether tics are totally involuntary (e.g., they also exist during sleep) or also have a voluntary tendency (e.g., to decide when to release a tic after an urge) has not been resolved yet. Hence, an investigation of involuntary vs. voluntary aspects of motor inhibition by means of TMS by measuring voluntary motor drive (VMD) could help to shed light on this issue. Heise et al. compared 14 healthy adolescents with 12 adolescents with TS (25). Youngsters with TS (including distal tics) showed a reduction of VMD, supporting the notion that tics could be seen as involuntary. This is underlined by studies in adults with lack of the Bereitschaftspotential (BP) before a tic (26).

Another issue regarding the motor network in TS is how patients initiate and guide their voluntary movements? Probably, the ability to suppress CSE ahead of volitional movements plays the major role in a normal performance and adolescents might have some problems achieving this. Draper et al. tested 10 adolescents with TS vs. healthy controls in a joint Go/No Go task with TMS (27). The main results of the MEP amplitude before volitional finger movements showed no differences between groups during the initial phase (0–60%) of movement preparation, but for the period immediately preceding the execution of a volitional movement, for the TS group there was no increase in MEP amplitude and no reduction in MEP variability. Individuals who exhibited the smallest increases in MEP amplitude (i.e., linear slope function) tended to show more severe motor tics, i.e., they were less able to modulate CSE. These findings are supported by similar results with TMS (MEP) in adults (TS: *n* = 11, HC: *n* = 11) (28) and children/adolescents (TS: *n* = 10; HC: *n* = 12) in Jackson et al. (29) with TS and suggest a stable long-term developmental mechanism of cortico-spinal hyper-excitability.

Brain dynamics of voluntary movements can also be registered using BP, which develops about 1,500 ms before the movement in question starts. In a free moving finger flexion task, Rothenberger et al. interpreted both the increased shift of BP amplitude from usual central to frontal sites in tic children and their less asymmetric lateralization as a sign of a different neuronal recruitment strategy and an expression of their need to invest more mental effort into control of a voluntary movement (TS: *n* = 10–22, HC: *n* = 10–22) (30–32). Since the neuronal generator of the BP is related to the supplementary motor area (SMA) which is connected to the cingulated gyrus, the authors suggest that this network must be involved in tic control quite early in life; an assumption which is confirmed by more recent studies in functional neuroimaging [see (33)].

Since eye blinking is a very frequent tic, Loo et al. decided to use the precise temporal resolution of the EEG (ERPs and spectral power) during cued voluntary eye blinks among children (8–12 years) with TS (*n* = 23) and without TS (*n* = 17) (34). Upon presentation of a cue every 3 s, children were instructed to make an exaggerated blink. The assumption was that differences between groups should show up in cortical areas typically associated with effortful control of eye blinks, i.e., frontal, premotor, parietal, occipital regions. It is important to know, that both groups of children had a 70% overlap of top regions with the highest dipole density, suggesting that similar cortical networks were used across groups to carry out the voluntary eye blink. However, children with TS exhibited several quantitative regional differences to healthy children with respect to spectral power within several oscillatory frequencies (theta, alpha, beta, gamma). Further, these differences were significantly associated with the severity and premonitory urge strength. The authors conclude “that children with chronic tic disorders exhibit abnormally high levels of neuronal activation used for regulation and effortful control of voluntary eye blinks.” This is in line with the notion that already elementary school children with TS have brain resources at their disposal which they can mobilize successfully directed to a certain goal.

Although all these reports favor the good abilities of children with TS for early and long-term compensation of their motor hyper-excitability, the question arises as to whether this still holds, when TS is associated with ADHD, what is often the case.

Reviewing physiological studies, Sukhodolsky et al. assumed that the frontal abnormalities (e.g., increased theta waves) as seen in children with ADHD, might limit their ability to mobilize this inhibitory system, while children with TS seem to be better off in this respect (35), which was already reflected in their higher fronto-central BP (TS + ADHD: *n* = 14, TS-only: *n* = 10) (32). Hence, TS children may use their preserved ability to mobilize frontal lobe activity also when both disorders are co-existent. This co-existence seems to follow an additive model with no interaction between factors and thus does not present as a new separate clinical entity (2). Moll et al. presented an informative motor example for additivity; they used TMS in children with TS, ADHD, TS plus ADHD and healthy controls (2 ×2 design with *n* = 16 in each group) (36, 37). For TS they reported (as expected) a shortened CSP, while for ADHD a reduced intracortical inhibition was found. In children with TS plus ADHD both abnormalities were registered providing evidence for an additive inhibitory deficit at the level of motor system excitability. However, further preliminary evidence of sufficient compensatory abilities (see above) in children with TS plus ADHD comes from a neurofeedback study by Gevensleben et al., where both tics and ADHD symptoms could be improved, probably on the basis of the neuronal resources related to TS (*n* = 46) (19).

## Sensory Processing

In children and adolescents, tics are sometimes (about 40%) preceded by premonitory sensory phenomena (PSP) such as an inner urge to move, or by bodily sensations such as feelings of mounting inner tensions or itching in certain areas of the body. Usually, they are transiently relieved by tic expression, which can be seen as a negative reinforcement of tics. In children, PSP is experienced rarely under the age of 10 years and is not a necessary prerequisite for a tic or its suppressibility, i.e., usually there is a de-coupling. In a sample of 254 outpatients with TS, 37% reported PSP while 64% were able to suppress their tics (38).

Electrophysiological studies in children and adolescents related to the issue of early sensory processing in TS are lacking. In adults, already in the 1980's somatosensory, visual and auditory evoked potentials, reflecting the basic functions of the respective sensory pathways, showed normal amplitudes and latencies up to about 100 ms after stimulus [(39); p. 138]. However, information processing thereafter may be deviated in TS (see paragraph on “cognition”).

Neurophysiological studies with MRI have shown some abnormalities in sensorimotor integration in TS such as thinning of the sensorimotor cortex in children and adolescents (40), which fits with the clinical observation in some adults of an increased sensitivity to stimuli. Specifically, altered sensorimotor gating was hypothesized as a core problem of TS. In an EMG/fMRI investigation of adolescents (TS: *n* = 22, HC: *n* = 22), Buse et al. (41) reported that the pre-pulse-inhibition (PPI) of the startle response was reduced in TS and related to brain regions other than the motor cortex, i.e., impaired intracortical inhibition may not be limited to the motor cortex (42). It was also hypothesized that in children with TS over- or under-responsiveness to environmental stimuli may play an important role; conceptualized as somatosensory dysregulation [SMD; (43)]. Sensory modulation was registered by self-report and, for example, von Frey filaments in the study by Weisman et al. who examined sensory threshold in children with TS plus comorbidities (*n* = 92; TS plus ADHD/depression/anxiety/obsessive-compulsive disorder). They found sensorimotor dysregulation in only 35% of cases, mostly related to the different comorbidities (43). Thus, more research is needed on sensorimotor gating before firm conclusions can be drawn (44).

## Cognition

The concept of non-voluntary neuronal disinhibition as the main pathophysiological background in TS along the lifetime is still a matter of debate (20). Specifically, this holds for qualitative and quantitative aspects during child development and how disinhibition can be counteracted/compensated via voluntary cognitive inhibitory processes. Roessner et al. (45, 46) and Greimel et al. (47) conducted several neuropsychological performance experiments in independent samples of children with TS, ADHD, TS plus ADHD and typically developing children (TDC) with about *n* = 20 in each group. In all, their empirical data clarified that a diagnosis of TS has little or no negative impact on neuropsychological performance and children with TS show normal response inhibition and strong executive functions. The evaluation of the four groups with a 2 ×2 factorial design (i.e., factor ADHD vs. factor TS) showed clearly that the factor ADHD was related to poor performance; there was no interaction between the two factors. This independency suggests an additive model for the association of TS and ADHD.

The favorable neuropsychological outcome of TS children could also be determined in the long-term by different groups [sample sizes from *n* = 14–29; 1-year follow-up in (48); 4.5 year follow-up in (49)].

In order to clarify the neurobiological background of these findings, electrophysiological correlates of cognitive control were investigated. Sensory information processing in the auditory domain with electrical brain activity indicators such as mismatch negativity (MMN) negative wave (Nd) and P300 in four groups of children (TDC, ADHD, ADHD plus CD, ADHD plus TS; *n* = 10/11 per group) revealed that neurodynamic sufficiency in ADHD and ADHD plus TS seems to be similarly impaired with ADHD as the responsible factor (50). Surprisingly, the above mentioned additive effect of TS and ADHD could not be found for event-related theta oscillations (ERT) in an auditory selective attention task, where ERT were enhanced in children with TS + ADHD only, supporting a joint effect [*n* = 53 for the four groups; (51)]. This held also for lower slow negative potential (SNP) amplitudes in an auditory S1-S2-RT paradigm [*n* = 10/11 per group; (52)]. In this task, the (aversive) S2 sound could be stopped by a button press initially (“normal condition”) followed by a task block where the sound could not be controlled any more without prior notice (“loss of control”). In a third block, the S2 stimulus was still not controllable (except in a few trials; “lack of control”). Analyzing the post-imperative-negative-variation (PINV), effects of TS and ADHD were revealed only in the non-control conditions, being independent in the lack-of-control condition, but interactive in the loss-of-control condition. Thus, the additive model of TS and ADHD could not be verified in the higher cognitive task condition of loss-of control (53). Taking these three studies together, they suggest that if very high cognitive performance is requested then the two associated disorders seem to interact at the level of electrophysiology, while at lower levels of cognitive demand the two factors may be separately but additively at work.

The additive model has also been supported by Shephard et al. (54). In a visual Go/NoGo task with registration of ERP measures (N2, P3, error-related negativity ERN, error positivity Pe) four groups of children (TS, ADHD, TS plus ADHD, TDC: *n* = 11–20 per group) were tested in a 2 ×2 factorial design. Only ADHD was associated with poorer performance and reduced amplitude of all ERPs, while TS was only related to slowed RTs, interpreted as a compensatory slowing of motor output to facilitate tic control. There was no interaction between TS and ADHD factors for any behavioral or ERP measure. The same authors conducted a similar four-group experiment on reinforcement learning (S-R; learn and reverse a stimulus-response-paradigm) (*n* = 13–20 per group) (55). As in the first experiment, there was no interaction between the two factors. The factor ADHD was responsible for impairment while TS children presented with intact learning and largely typical ERP amplitudes (stimulus-locked P3, feedback-locked P2, feedback-learned negativity FRN). These results fit with an additive model for TS plus ADHD.

Similarly, favorable results for TS were reported in the long-term. For example, Roessner et al. found stable normal executive function performance in TS after 1-year follow-up (48). Eichele et al., using a modified Erikson flanker task in children with TS, ADHD and TDC performing two testing sessions administered on average 4.5 years apart (at t1: 10 years; at t2: 14.5 years) found that all groups improved with task performance and ERPs (i.e., P300 and ERN amplitude) over time, but only the TS group trajectories converged to normal with maturation (49). ERP component amplitudes correlated with worst-ever tic scores. The changes are consistent with the development of cognitive compensatory self-regulation mechanisms and spontaneous inhibitory maturation of the brain during early years of adolescence [see also TMS data in (23)]. This may best explain the improvement in tic suppression/decrease during these years and may support the application of cognitive-behavioral-therapy (CBT) in order to nurture these maturational brain dynamic processes.

Within this context of inhibitory control of tics, its probable modulation via external sensory stimuli and their processing on the way to a motor response deserves a closer look. Petruo et al., examined adolescents with TS (TS: *n* = 35, HC: *n* = 39) in a Go/NoGo task. While testing for the inhibition of a motor response they manipulated the complexity of sensory/stimulus input (i.e., trigger either unimodal visual or bimodal auditory-visual). They concluded that the binding within the sequence of stimulus perception-motor impulse-inhibition is stronger in TS and thus may reflect a strengthened perception-action integration (56). However, a control group with ADHD was lacking, questioning whether either the factor tic or ADHD was responsible for this effect. On the other hand, comprehensive behavioral intervention (CBIT) could normalize perception-action binding in TS (i.e., improving inhibitory control) (TS: *n* = 21, HC: *n* = 21) (57) and analyses of EEG in adults with TS (TS: *n* = 24 TS, HC: *n* = 24; visual-motor event file task requiring repeating or alternating responses) suggests that motor and sensory processes seem to be less relevant but merely cognitive processes may be important for linking perception to action (58).

## Sleep

Tics can be observed also during sleep and different kind of sleep problems are well-known to be associated with TS. A still valid overview on sleep and TS is provided by Rothenberger et al. (59), who stated that polysomnographic sleep parameters in TS are disturbed (e.g., decreased sleep efficiency, enhanced wakefulness after sleep onset, increased arousal phenomena, decreased REM sleep). Table 1 summarizes the main findings regarding the different domains described in this and the previous sections.

**Table 1:** Electrophysiological findings in children and adolescents with Tourette syndrome.

**Domains**	**Findings and Interpretations**
Basic electrical brain activity (oscillations)	• Minor irregularities in the resting EEG (visual inspection) in about half of the patients (11) • Decreased intrinsic long-range connectivity between the frontal and the temporal/occipital/parietal lobes (14) • Misguided thalamo-cortical brain oscillations (18)
Motor aspects	• Shortened TMS-induced cortical silent period indicating deficient inhibitory motor control; compatible with tic generation at the level of the basal ganglia (21); developmental effect re. tic presence in target muscle (23) • Less modulation of cortico-spinal excitability in go/nogo task with increasing tic severity (27) • More frontal and less lateralized distribution of the Bereitschaftspotential (BP) (30–32); use of similar networks but quantitative differences (higher activation) during voluntary blinking (34); more effortful control of voluntary movements
Sensory processing	• No differences in early ERP components (latencies <100 ms) • Reduced pre-pulse inhibition of the startle response indicating deficient sensorimotor gating (42) • Sensorimotor dysregulation primarily related to comorbid ADHD (44)
Cognition	• Normal response inhibition/executive functions (neuropsychological test performance), also in the long-term (45–49) • Stronger perception action-binding due to cognitive processes linked to the inferior parietal cortices (56, 58)
Sleep	• Disturbed sleep parameters (e.g., decreased sleep efficiency increased arousal phenomena) (59) indicating sleep hygiene as part of a treatment program
Comorbid ADHD	• Mainly additive effects (exception: high cognitive demands?), more aberrations with consequences for treatment

*Only findings from studies are listed with at least 20 youngsters with TS*.

In the 1990's, Drake et al. used a four-channel cassette EEG sleep recording in 20 TS patients (age 10–36 years) in their usual sleeping environments and routines (60). They detected that chronic tics may persist in sleep and may cause awakenings and thus fragmentation of sleep, including reduced REM sleep. However, in later empirical studies with better electrophysiological equipment (multi-channel sleep polysomnography in sleep laboratories) REM sleep was found normal in TS throughout development, while some of the other findings such as increased short arousals could be confirmed. The latter may be intrinsic to TS from its onset in childhood and might trigger tics and other behavioral problems during the day [e.g., increased stress-sensitivity, daytime sleepiness; (61–64)] When ADHD symptoms accompany TS, also increased motor restlessness and disorganized behavior might take place (*n* = 90; four groups: TD, ADHD, TD + ADHD, HC) (65).

In summary, in children with TS plus ADHD sleep problems may be higher, having both REM sleep increase (for ADHD) and lower sleep efficiency/elevated arousal (for TS) in an additive manner, without indicating any interaction effect [*n* = 18 in each of the four groups in (63); TD + ADHD: *n* = 19; HC: *n* = 19 HC in (66)]. Thus, sleep hygiene should be part of a TS treatment program.

## Clinical Applications

Deviations of electrophysiological measures, either as a sign of defective inhibition (e.g., brain oscillations, TMS-MEP, ERPs) or its compensation (e.g., BP, brain oscillations, ERPs) reflect a clinical-physiological correlation that could be used for treatment approaches for TS intended to improve automatic and/or cognitive control of tics (Table 2). Also, these tools could be used to control for adverse effects of psychopharmacotherapy [e.g., (67)].

**Table 2:** Clinical applications based on electrophysiology in children and adolescents with Tourette syndrome.

Tic reduction *via* neuromodulatory approaches targeting neuronal deficits and/or strengthening compensatory brain mechanisms:
• endogenous: e.g., neurofeedback • exogenous: e.g., repetitive transcranial magnetic stimulation (rTMS), transcranial direct current stimulation (tDCS)
Predictors for treatment outcome

*So far, only first preliminary studies have been conducted*.

The importance of dysrhythmic brain oscillations in TS has already been clarified [e.g., (17, 18)] Hence, improving rhythmicity/synchronization of brain oscillations along the striatal-thalamic-cortical circuit by voluntary training could be promising, specifically, when co-existing ADHD symptoms are taken into consideration. This would also allow improvement of executive control functions via increasing attentional capacities (68). Neurofeedback (NF) seems to be a good candidate. During NF, specific aspects of brain activity of the patient [e.g., theta-beta ratio; slow cortical potential (SCP) amplitudes] is fed-back online using visual or auditory signals, allowing the participant to learn how to actively train his brain to gain control over these otherwise unaware neuronal processes and thus his behavior (4, 69). A successful preliminary clinical study in children with tic disorders encourages this idea (13): Both NF protocols led to a reduction of tic frequency (ca. 20%) after about 20 training units while SCP NF also had positive effects on associated ADHD symptomatology.

Other, merely passive electrophysiological treatment options are rTMS and tDCS, which appear to be safe for children and adolescents, although there remain many unmet needs in pediatric patients. Further, NF, rTMS, and tDCS are not disorder specific but rather may help to modulate neuronal excitability within the regional network stimulated on the scale of minutes to hours and might have clinical effects that can last weeks to months (70).

For example, Kwon et al. designed a pilot, open-label 12 weeks cohort study to assess efficacy of low-frequency rTMS (SMA stimulation, 1Hz, 100% motor threshold, 1,200 stimuli/day) in 10 male children with TS (11.2 years) (71). Tic symptoms improved significantly with no side effects. Also, Le et al. used a similar open label stimulating procedure in 25 children with TS (aged under 16 years) over 4 weeks and reported a significant reduction of tics and ADHD symptoms which was stable at 6 months follow-up (72). Beyond rTMS at SMA, Wu et al. brought Theta-Burst-TMS (TBS) in children into treatment discussion (73). They applied a single session of TBS over the left motor cortex to 16 children with TS (mean 12 years) and 24 HC (mean 12 years) and found the method safe and well-tolerated; effects on tics were not reported. The same group conducted a preliminary randomized, double-blind sham-controlled TBS-SMA/fMRI study (8 sessions over 2 days) using a trial in 12 patients with TS (10–22 years old; comorbid ADHD, OCD, and concurrent medication) and did not find a larger tic reduction following TBS compared to the sham-condition (74).

The therapeutic potential of tDCS (stimulation over the left DLPFC) in children and adolescents with mental disorders is limited but has been tested in autism spectrum disorder (two studies; total *n* = 40) and ADHD (three studies; total *n* = 43) indicating some improved inhibitory control (70). The only sham-controlled study of cathodal tDCS (single-session) with a group of 10 young adults with TS (two of the patients were between 16–18 years) was conducted by Dyke et al. (75). They applied tDCS to the SMA and measured pre-post changes in cortical excitability using single-pulse TMS over the primary motor cortex (M1). Also, pre-post video recordings of tics were taken. There was no indication of a statistically significant change in M1 cortical excitability, but tic impairment scores improved. However, the authors themselves saw several methodological weaknesses in their study such as high individual variability in response to stimulation. Hopefully, individualized protocols and longer-term studies might further develop the therapeutic potential of tDCS with its advantage in being safe, cheap, portable and easy to administer at home. However, without further studies including clinically well-characterized and large enough samples and using sham control conditions one should be hesitant to interpret the practical value of rTMS and tDCS in TS.

In summary, neuromodulation *via* electrophysiological means may pave a new avenue of treatment in youngsters with developmental neuropsychiatric disorders, although the excitability characteristics of the developing brain pose challenges.

## Conclusions and Perspectives

Applying electrophysiological approaches, a comprehensive picture of developmental brain dynamics in children and adolescents with Tourette syndrome could be revealed showing that electrophysiological aspects do not only echo neuronal processes but also parallel clinical characteristics of TS. There is clear brain electrical evidence in TS for a maturational delay/deficit of motor inhibition with cortico-spinal hyper-excitability which is assumed to be successfully counteracted/compensated by voluntary cognitive inhibitory processes; comparable in strength to the level of typically developing children.

To gain further insight into the neurobiological/ neurodynamic background of Tourette syndrome, it may be helpful to study the interplay of neuronal processes by combining different approaches, for example electrical brain activity and functional magnetic resonance imaging (fMRI).

The comorbidity of Tourette syndrome and ADHD has been considered systematically (i.e., applying a 2 ×2 design) in only part of the studies. Thus, it remains open for some of the findings whether they rather reflect the factor tic or ADHD. Generally, effects indicate an additive model, i.e., an independent contribution of each factor to the electrophysiological parameter in question. Only, in the context of (very) high cognitive demands, do the two associated disorders appear to interact functionally.

Neuromodulation via electrophysiological means (e.g., neurofeedback, rTMS and tDCS) aim at targeting neuronal deficits and/or strengthening compensatory mechanisms. Pilot studies have been conducted in children and adolescents with Tourette syndrome, some with a probable reduction in tic severity. Further, well-controlled studies with larger samples and longer-term outcomes are necessary before firm conclusions can be drawn regarding the clinical benefit but also risks of these approaches for the developing brain.

Other challenges for the future are the prediction of the course of the disorder for an individual patient and, in times of “personalized medicine,” the identification of predictors for treatment outcome. In this respect, electrophysiological measures may represent promising markers as has already been reported for ERP components regarding neurofeedback in children and adolescents with ADHD or cognitive-behavioral therapy in adult patients with Tourette syndrome (76).

In conclusion, application of electrophysiology in youngsters with TS seems to have been promising for recent clinical and research aspects and is expected to remain promising for the future.

## Author Contributions

AR initiated this review and drafted the manuscript. HH contributed to the writing of the manuscript. All authors approved the final manuscript.

## Conflict of Interest

HH is an employee of neuroCare Group. The remaining author declares that the research was conducted in the absence of any commercial or financial relationships that could be construed as a potential conflict of interest.
